# Cure a La Astral

**Published:** 1915-01

**Authors:** 


**Cure a La Astral** (sic.) W. Stuart Leech, Roseau, Minn., Wis. Med. Recorder, Sept. 1914. At first reading, this article seemed to be either figurative or a bit of sarcasm but it concludes with a positive statement of result. The case was that of a boy with symptoms of appendicitis, with fear of pus, temperature as high as 102.5. The evening before the physical consultation, three physicians went into the Desire World (dreamland) and met at the bed-side of the patient—invisibly, being in the astral plane. One of them seized the offending organ and threw it away, another massaged the colon. At the physical examination, the boy appeared well and showed no subsequent symptoms for six months. This report, in the twentieth century, in the U. S., is certainly interesting.

